# Plasma Phosphorylated Tau 217 and Incident Mild Cognitive Impairment and Dementia in Older Women

**DOI:** 10.1001/jamanetworkopen.2026.1295

**Published:** 2026-03-10

**Authors:** Aladdin H. Shadyab, Bowei Zhang, Andrea Z. LaCroix, Michelle M. Mielke, Susan M. Resnick, Steve Nguyen, Luigi Ferrucci, Towia A. Libermann, Long Ngo, Ramon Casanova, Alexander P. Reiner, Danni Li, Caroline M. Nievergelt, Adam X. Maihofer, JoAnn E. Manson, Linda K. McEvoy

**Affiliations:** 1 Herbert Wertheim School of Public Health and Human and Human Longevity Science, University of California San Diego, La Jolla; 2 Division of Geriatrics, Gerontology, and Palliative Care, Department of Medicine, University of California San Diego, La Jolla; 3 Department of Epidemiology and Prevention, Wake Forest University School of Medicine, Winston-Salem, North Carolina; 4 Laboratory of Behavioral Neuroscience, National Institute on Aging, Bethesda, Maryland; 5 Longitudinal Studies Section, Translational Gerontology Branch, National Institute on Aging, Bethesda, Maryland; 6 Division of Interdisciplinary Medicine and Biotechnology, Beth Israel Deaconess Medical Center, Boston, Massachusetts; 7 Beth Israel Deaconess Medical Center Genomics, Proteomics, Bioinformatics, and Systems Biology Center, Boston, Massachusetts; 8 Harvard Medical School, Boston, Massachusetts; 9 Division of General Medicine, Department of Medicine, Beth Israel Deaconess Medical Center, Boston, Massachusetts; 10 Department of Biostatistics, Harvard T.H. Chan School of Public Health, Boston, Massachusetts; 11 Department of Biostatistics and Data Science, Wake University School of Medicine, Winston-Salem, North Carolina; 12 Department of Epidemiology, University of Washington, Seattle; 13 Department of Laboratory Medicine and Pathology, University of Minnesota, Minneapolis; 14 Department of Psychiatry, University of California San Diego, La Jolla; 15 Division of Preventive Medicine, Department of Medicine, Brigham and Women’s Hospital, Harvard Medical School, Boston, Massachusetts; 16 Department of Epidemiology, Harvard T.H. Chan School of Public Health, Boston, Massachusetts; 17 Kaiser Permanente Washington Health Research Institute, Seattle, Washington

## Abstract

**Importance:**

There is limited research on the long-term associations of plasma phosphorylated tau 217 (p-tau217) with mild cognitive impairment (MCI) and dementia. No study has evaluated whether such associations vary by race or hormone therapy (HT) use.

**Objective:**

To examine associations of baseline plasma p-tau217 with incident MCI and dementia and determine whether associations vary by age, race, *APOE* ε4 carrier status, or HT use.

**Design, Setting, and Participants:**

This cohort study examined women recruited from 39 US clinical sites between 1996 and 1999 into the Women’s Health Initiative Memory Study who were randomized to either estrogen alone vs placebo or estrogen plus progestin vs placebo. Women were assessed for up to 25 years through 2021. Baseline plasma p-tau217 was measured in 2024 and analyzed between February and August 2025. Women aged 65 to 79 years who were cognitively unimpaired at baseline were included for this analysis.

**Exposure:**

Plasma p-tau217, quantified using the ALZpath Simoa assay.

**Main Outcomes and Measures:**

The primary outcome was the combined end point of incident MCI or probable dementia. Secondary outcomes included MCI and dementia examined separately. Cause-specific hazard ratios (HRs) and 95% CIs for the association of p-tau217 with MCI or dementia were estimated using Cox proportional hazards regression models.

**Results:**

Among 2766 participants (mean [SD] age, 69.9 [3.8] years; 486 [17.9%] Black, 196 [7.1%] Hispanic, and 2007 [73.9%] White), 1311 developed the combined end point of MCI or dementia (849 participants with MCI and 752 participants with dementia). Every 1-SD increase in log_2_-transformed p-tau217 was associated with incident MCI or dementia (HR, 2.43; 95% CI, 2.18-2.71) and each individual outcome (MCI: HR, 1.94; 95% CI, 1.72-2.20; dementia: HR, 3.17; 95% CI, 2.79-3.61). Associations of p-tau217 with dementia were larger in magnitude for women randomized to estrogen plus progestin (HR, 4.18; 95% CI, 3.41-5.13) vs placebo (HR, 3.07; 95% CI, 2.41-3.91) (*P* for interaction = .04) but did not significantly vary by estrogen alone vs placebo. P-tau217 associations with MCI or dementia were larger in magnitude for women older than 70 years (*P* for interaction = .04), *APOE* ε4 carriers (*P* for interaction = .02), and White women compared with Black women (*P* for interaction < .001). However, the combination of p-tau217 and age performed similarly in White women (area under the curve = 72.0%; 95% CI, 70.3%-73.6%) and Black women (area under the curve = 70.4%; 95% CI, 64.0%-78.0%). P-tau217 was not associated with incident MCI in Black women.

**Conclusions and Relevance:**

In this cohort study of cognitively unimpaired older women, p-tau217 was associated with incident MCI or dementia up to 25 years later. These findings suggest that age, race, *APOE* ε4, and HT use should be considered when examining associations of p-tau217 with cognitive outcomes.

## Key Points

**Question:**

Do associations of plasma phosphorylated tau 217 (p-tau217) with incident mild cognitive impairment (MCI) and dementia vary by race, hormone therapy, age, or *APOE* ε4 carrier status?

**Findings:**

In this cohort study among 2766 older women, associations of p-tau217 with incident dementia were larger in magnitude among women assigned to estrogen plus progestin vs placebo but did not vary for estrogen alone vs placebo. P-tau217 associations with MCI or dementia were larger in magnitude for women older than 70 years, *APOE* ε4 carriers, and White compared with Black women.

**Meaning:**

These findings underscore the value of p-tau217 and show that many factors should be considered when examining its associations with cognitive outcomes.

## Teaser

This cohort study examines associations of baseline plasma phosphorylated tau 217 with incident mild cognitive impairment (MCI) or dementia and whether associations vary by age, race, *APOE* ε4 carrier status, or hormone therapy use among older men and women.

## Introduction

Plasma biomarkers of Alzheimer disease (AD) offer a minimally invasive, accessible approach for detecting AD pathology.^1^ Plasma phosphorylated tau 217 (p-tau217) has demonstrated higher accuracy in detecting AD pathology relative to other biomarkers,^2,3,4,5^ with equivalent performance as cerebrospinal fluid p-tau217 for AD diagnosis.^6^

Plasma p-tau217 has been associated with incident dementia in community samples.^7,8,9,10^ However, several gaps in knowledge remain. Few studies have examined associations of p-tau217 with incident mild cognitive impairment (MCI) and dementia in community-based cohorts of cognitively healthy individuals with more than 2 decades of follow-up; prior studies had follow-up periods of 4 to 16 years.^7,8^ Racial differences in the discriminative accuracy of p-tau217 for MCI or dementia are not well understood, despite known racial disparities in dementia risk.^7,8,11,12^ Few studies have evaluated p-tau217 in Black adults.^13,14,15,16^ Although self-reported hormone therapy (HT) has been associated with increased tau accumulation, amyloid deposition, and dementia in observational studies,^17,18,19,20,21^ no study has examined whether the association of p-tau217 with cognitive outcomes differs by randomized assignment to HT.

The Women’s Health Initiative Memory Study (WHIMS) is the only large randomized clinical trial examining the effects of HT on cognitive outcomes among postmenopausal women.^22,23^ In this cohort study of WHIMS participants, we examined associations of baseline plasma p-tau217 with incident MCI or dementia for up to 25 years of follow-up and evaluated whether associations varied by age, race, *APOE* ε4, or randomization to HT.

## Methods

### Study Design and Population

This cohort study was approved by the institutional review board of Fred Hutchinson Cancer Center and followed the Strengthening the Reporting of Observational Studies in Epidemiology (STROBE) reporting guideline. WHIMS included 2 randomized clinical trials investigating the effects of HT on cognitive outcomes among 7479 community-dwelling, cognitively unimpaired postmenopausal women aged 65 to 79 years.^22,23^ Women were recruited from 39 US clinical centers from 1996 to 1999 and randomized to oral conjugated equine estrogens (0.625 mg per day) vs placebo (estrogen alone vs placebo) among those with hysterectomy or conjugated equine estrogens combined with medroxyprogesterone acetate (2.5 mg per day) vs placebo (estrogen plus progestin vs placebo) for those with an intact uterus. Randomization was determined using a permuted block algorithm stratified by age group and clinical center site, which eliminated confounding due to site differences. The trials were stopped in 2004 and 2002, respectively; annual in-person follow-up continued through 2007. In 2008, WHIMS transitioned to annual telephone-administered cognitive assessments, which followed participants for cognitive outcomes through 2021.^24^ Among the 7479 WHIMS participants, we selected 2836 for biomarker measurement, including all 1334 women with incident MCI or probable dementia and a subset of 1502 controls who did not develop these outcomes during follow-up (eMethods and eFigure 1 in Supplement 1). All participants provided written informed consent.

### Plasma P-Tau217 Measurement

Fasting blood was drawn at baseline, processed, frozen at −70 °C, and stored in a repository in Rockville, Maryland (Fisher Bioservices). Samples were shipped in 2024 to the Advanced Research and Diagnostic Laboratory at University of Minnesota on dry ice for p-tau217 measurement using the ALZpath Simoa pTau-217 v2 assay (eMethods in Supplement 1). Samples were assayed in singlets with the inclusion of 192 duplicates; laboratory personnel were blinded to the inclusion of duplicates and cognitive impairment status. The average intraassay coefficient of variation derived from duplicates was 11.4%.

### Outcomes

Our primary outcome was the combined end point of MCI or dementia. We also evaluated each individual outcome separately in secondary analyses. MCI and dementia were ascertained and adjudicated annually (eMethods in Supplement 1). MCI was based on the Petersen criteria, and dementia was based on *Diagnostic and Statistical Manual of Mental Disorders* (Fourth Edition) criteria.^25,26^

### Covariates

Participants completed baseline questionnaires assessing self-reported race (American Indian or Alaska Native, Asian, Black, Native Hawaiian or Other Pacific Islander, White, more than 1 race, or missing) and ethnicity (Hispanic or Latino, not Hispanic or Latino, or missing), age, education, smoking status, treated diabetes, cardiovascular disease, and total energy expenditure from recreational physical activity. Hypertension was defined as either self-report of physician-diagnosed hypertension, use of hypertensive medications, systolic blood pressure of 130 mm Hg or greater, or diastolic blood pressure of 80 mm Hg or greater, measured at the clinic visit. Body mass index was calculated as weight in kilograms divided by height in meters squared. The WHI dataset contained *APOE* e4 genotype for White women only based on 2 single nucleotide polymorphisms, rs429358 and rs7412. Other covariates included HT treatment group, total cholesterol, high-density lipoprotein cholesterol, and estimated glomerular filtration rate (eGFR), calculated based on serum creatinine using the 2021 Chronic Kidney Disease Epidemiology Collaboration equation.^27^

### Statistical Analysis

P-tau217 was log_2_ transformed and standardized; we excluded 2 outliers, defined as values more than 5 SDs above the mean. Continuous covariates were compared across quartiles of p-tau217 using Kruskal-Wallis tests. Categorical variables were compared using χ^2^ tests or Fisher exact test.

We estimated cause-specific hazard ratios (HRs) and 95% CIs for the association of p-tau217 with MCI or dementia using Cox proportional hazards regression models with robust standard errors. Models were weighted according to inverse probability and sampling weights to account for the sampling design (eMethods in Supplement 1). Women were followed from the date of WHIMS randomization to the date of the cognitive assessment that triggered the first diagnosis of MCI or dementia, loss to follow-up, or November 3, 2021 (end of study), whichever came first. Women who did not develop MCI or dementia, including those who died or ended the study for other reasons, were censored at the date of their final cognitive assessment, consistent with prior WHIMS publications.^22,23^ Models were adjusted for the previously mentioned covariates, selected according to prior literature.^28^ We did not adjust for *APOE* ε4 because this variable was available in White women only, and adjustment did not materially change findings. The proportional hazards assumption was assessed using Schoenfeld residual plots and log-log survival plots; no violations were observed. To account for missing covariate data, we applied multiple imputation by chained equations, specifying all study variables with 20 imputations and 20 iterations. We repeated the analyses using MCI and dementia as individual outcomes of interest.

We evaluated subgroup differences by race, age, HT, and *APOE* ε4, which were determined a priori. Differences by race were focused on Black and White women, given smaller sample sizes in other races. Interaction terms between these factors and p-tau217 were included in the models.

We performed several sensitivity analyses. First, we evaluated associations of p-tau217 quartiles with the outcomes to identify potential nonlinear associations. Second, we excluded those with eGFR of 60 mL/min/1.73 m^2 ^or less because chronic kidney disease and low eGFR are associated with elevated plasma AD biomarker levels, even in the absence of AD neuropathology.^29,30^ Third, we repeated analyses accounting for competing risk of death using Fine-Gray models, adjusting for the same covariates. In the Fine-Gray models, death prior to MCI or dementia was treated as a competing event using the actual date of death, whereas women who were alive and free of MCI or dementia were censored at their last cognitive assessment. We also generated weighted cumulative incidence curves for the combined outcome of MCI or dementia or dementia according to p-tau217 quartiles, accounting for competing risk of death.

We assessed the discriminative accuracy of p-tau217 using time-dependent receiver operating characteristic (ROC) curves generated from Cox models at the median follow-up. We determined sensitivity and specificity for p-tau217 alone and combined with age, race, and ethnicity.

Analyses were conducted from February to August 2025 using R version 4.4.3 (R Project for Statistical Computing). Time-dependent ROC curves were generated using the risksetROC package in R. Tests were 2-sided. Significance was defined as 95% CIs that did not cross 1 and *P* < .05. Analyses of secondary outcomes were considered hypothesis generating. Because we tested for multiple interactions, subgroup differences may be due to chance and should be interpreted cautiously.

## Results

The analytic sample included 2766 women (eFigure 1 and eTable 1 in Supplement 1). The mean (SD) baseline age was 69.9 (3.8) years, and 17 (0.6%) were American Indian or Alaska Native, 121 (4.5%) were Asian, 486 (17.9%) were Black, 196 (7.1%) were Hispanic or Latino, 8 (0.3%) were Native Hawaiian or Other Pacific Islander, 2007 (73.9%) were White, and 75 (2.8%) were more than 1 race (Table). Women with higher levels of baseline p-tau217 were more likely to be older, White, have lower body mass index, have never smoked, have lower eGFR, and be *APOE* ε4 carriers; there were no other differences (Table). Differences in baseline characteristics between Black and White women are shown in eTable 2 in Supplement 1. P-tau217 was lower on average in Black vs White women (eFigure 2 in Supplement 1). During a median (range) follow-up of 14.1 (0.9-25.2) years, 1311 participants developed the combined end point of MCI or dementia (849 developed MCI and 752 developed dementia).

**Table.** Baseline Characteristics by Baseline Plasma P-Tau217, Women’s Health Initiative Memory Study, 1996-1999

Characteristic	Participants by quartile of p-tau217, No. (%)					*P* value
	Overall (N = 2766)	Quartile 1: 0.014-0.230 pg/mL (n = 690)	Quartile 2: 0.230-0.305 pg/mL (n = 687)	Quartile 3: 0.305-0.438 pg/mL (n = 695)	Quartile 4: 0.438-2.564 pg/mL (n = 694)	
Age, mean (SD), y	69.9 (3.8)	68.8 (3.3)	69.2 (3.5)	70.3 (3.8)	71.1 (4.0)	<.001
Hormone therapy treatment group						
Estrogen alone placebo	569 (20.6)	144 (20.9)	144 (21.0)	145 (20.9)	136 (19.6)	.30
Estrogen alone intervention	570 (20.6)	166 (24.1)	138 (20.1)	123 (17.7)	143 (20.6)	
Estrogen plus progestin placebo	803 (29.0)	187 (27.1)	207 (30.1)	209 (30.1)	200 (28.8)	
Estrogen plus progestin intervention	824 (29.8)	193 (28.0)	198 (28.8)	218 (31.4)	215 (31.0)	
Race						
American Indian or Alaska Native	17 (0.6)	7 (1.0)	2 (0.3)	5 (0.7)	3 (0.4)	<.001
Asian	121 (4.5)	33 (4.9)	38 (5.6)	31 (4.6)	19 (2.8)	
Black	486 (17.9)	162 (23.8)	116 (17.2)	114 (16.7)	94 (13.8)	
Native Hawaiian or Other Pacific Islander	8 (0.3)	2 (0.3)	1 (0.1)	4 (0.6)	1 (0.1)	
White	2007 (73.9)	451 (66.3)	500 (74.3)	514 (75.5)	542 (79.7)	
>1 Race	75 (2.8)	25 (3.7)	16 (2.4)	13 (1.9)	21 (3.1)	
Missing, No.	52	10	14	14	14	NA
Ethnicity						
Hispanic or Latino	196 (7.1)	55 (8.1)	51 (7.5)	50 (7.2)	40 (5.8)	.42
Not Hispanic or Latino	2548 (92.9)	628 (91.9)	631 (92.5)	641 (92.8)	648 (94.2)	
Missing, No.	22	7	5	4	6	NA
Body mass index, mean (SD)^a^	28.6 (5.6)	29.2 (5.6)	28.6 (5.6)	28.5 (5.6)	28.0 (5.7)	<.001
Missing, No.	13	3	6	2	2	NA
Smoking status						
Never smoked	1556 (57.1)	359 (53.0)	372 (54.8)	412 (60.2)	413 (60.2)	<.001
Past smoker	1024 (37.6)	262 (38.7)	283 (41.7)	237 (34.6)	242 (35.3)	
Current smoker	146 (5.4)	56 (8.3)	24 (3.5)	35 (5.1)	31 (4.5)	
Missing, No.	40	13	8	11	8	NA
Education						
Less than high school equivalent	256 (9.3)	67 (9.7)	66 (9.6)	67 (9.7)	56 (8.1)	.13
High school diploma or GED	609 (22.1)	140 (20.3)	165 (24.1)	158 (22.9)	146 (21.1)	
Some college or associate	1031 (37.4)	272 (39.5)	238 (34.7)	234 (33.9)	287 (41.5)	
College graduate or higher	861 (31.2)	210 (30.5)	217 (31.6)	232 (33.6)	202 (29.2)	
Missing, No.	9	1	1	4	3	
Diabetes						
Overall	190 (6.9)	47 (6.8)	45 (6.6)	50 (7.2)	48 (6.9)	.97
Missing	6	1	1	1	3	NA
Cardiovascular disease	134 (4.8)	34 (4.9)	29 (4.2)	35 (5.0)	36 (5.2)	.84
Physical activity						
Mean (SD) MET h/wk	11.5 (13.7)	11.4 (13.0)	11.8 (14.2)	10.9 (12.7)	12.2 (14.8)	.65
Missing	8	4	2	0	2	NA
Total cholesterol						
Mean (SD), mg/dL	234.2 (39.5)	233.5 (39.6)	236.0 (40.4)	233.8 (40.2)	233.4 (38.1)	.66
Missing	429	109	108	109	103	NA
HDL cholesterol						
Mean (SD), mg/dL	53.6 (12.4)	52.7 (12.0)	53.2 (11.8)	53.9 (13.5)	54.5 (12.3)	.05
Missing	429	109	108	109	103	
eGFR						
Mean (SD), mL/min/1.73 m^2^	83.7 (13.5)	86.9 (12.0)	83.9 (12.6)	82.4 (13.3)	81.8 (15.3)	<.001
Missing, No.	430	109	109	109	103	NA
*APOE* e4 genotype						
Noncarrier	1330 (73.2)	342 (84.4)	369 (82.2)	346 (74.4)	273 (54.9)	<.001
Carrier	486 (26.8)	63 (15.6)	80 (17.8)	119 (25.6)	224 (45.1)	
Missing, No.	950	285	238	230	197	NA
Hypertension						
Overall	1948 (70.7)	495 (71.8)	469 (68.7)	491 (71.2)	493 (71.0)	.59
Missing, No.	10	1	4	5	0	NA

Abbreviations: eGFR, estimated glomerular filtration rate; GED, general educational development; HDL, high-density lipoprotein; MET, metabolic equivalent; NA, not applicable; p-tau217, phosphorylated tau 217.

SI Conversions: To convert total and HDL cholesterol to millimoles per liter, multiply by 0.0259.

^a^ Calculated as weight in kilograms divided by height in meters squared.

**Figure 1.**
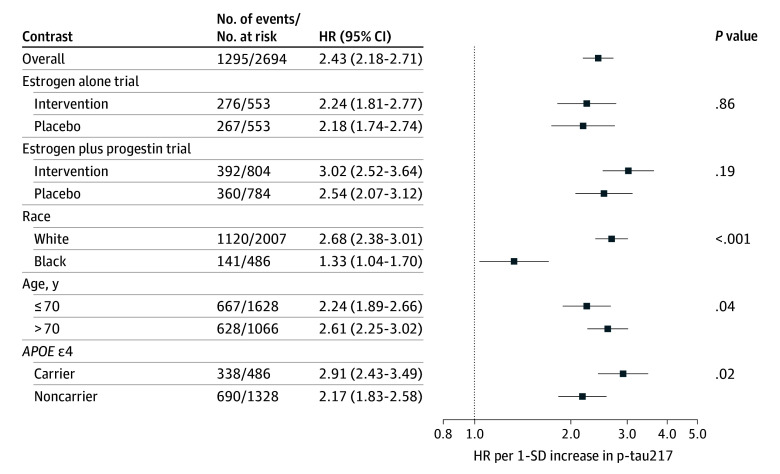
Forest Plot of the Association of Baseline Plasma Phosphorylated Tau 217 (P-Tau217) With Mild Cognitive Impairment or Dementia Hazard ratios (HRs) and 95% CIs were derived from Cox proportional hazards regression models. All models were adjusted for hormone therapy trial group, age, race, ethnicity, education, body mass index, smoking status, diabetes, cardiovascular disease, hypertension, physical activity, estimated glomerular filtration rate, total cholesterol, and high-density lipoprotein cholesterol. Models were weighted to account for biomarker sample selection. The *P* value for interaction modeled age as a continuous variable. The analytic sample included 2694 women after removing those with missing race or ethnicity. The number of events per number at risk for the unweighted sample is shown.

**Figure 2.**
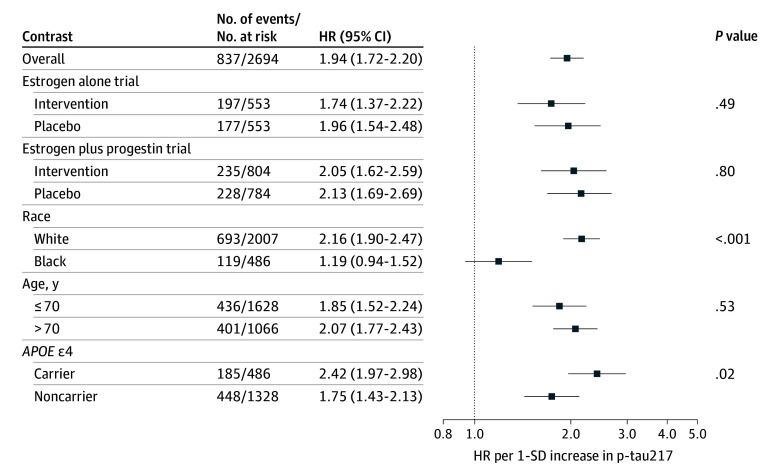
Forest Plot of the Association of Baseline Plasma Phosphorylated Tau 217 (P-Tau217) With Incident Mild Cognitive Impairment Hazard ratios (HRs) and 95% CIs were derived from Cox proportional hazards regression models. All models were adjusted for hormone therapy trial group, age, race, ethnicity, education, body mass index, smoking status, diabetes, cardiovascular disease, hypertension, physical activity, estimated glomerular filtration rate, total cholesterol, and high-density lipoprotein cholesterol. Models were weighted to account for biomarker sample selection. The *P* value for interaction modeled age as a continuous variable. The analytic sample included 2694 women after removing those with missing race or ethnicity. The number of events per number at risk for the unweighted sample is shown.

**Figure 3.**
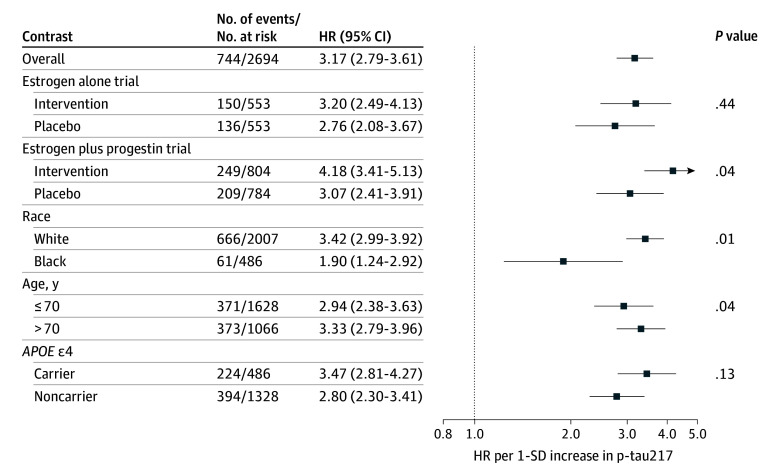
Forest Plot of the Association of Baseline Plasma Phosphorylated Tau 217 (P-Tau217) With Incident Dementia Hazard ratios (HRs) and 95% CIs were derived from Cox proportional hazards regression models. All models were adjusted for hormone therapy trial group, age, race, education, ethnicity, body mass index, smoking status, diabetes, cardiovascular disease, hypertension, physical activity, estimated glomerular filtration rate, total cholesterol, and high-density lipoprotein cholesterol. Models were weighted to account for biomarker sample selection. The *P* value for interaction modeled age as a continuous variable. The analytic sample included 2694 women after removing those with missing race or ethnicity. The number of events per number at risk for the unweighted sample is shown.

Associations of p-tau217 with the outcomes are shown in Figure 1, Figure 2, and Figure 3. In the fully adjusted model, higher p-tau217 was associated with incident MCI or dementia (HR, 2.43; 95% CI, 2.18-2.71) (Figure 1). When examining each outcome separately, the largest magnitude of association was observed for dementia (HR, 3.17; 95% CI, 2.79-3.61) (Figure 3). The association was lower in magnitude but still significant when examining MCI (HR, 1.94; 95% CI, 1.72-2.20) (Figure 2).

The association of p-tau217 with cognitive outcomes did not significantly differ by estrogen alone vs placebo (Figure 1, Figure 2, and Figure 3). The association of p-tau217 with dementia was larger in magnitude for estrogen plus progestin (HR, 4.18; 95% CI, 3.41-5.13) compared with placebo (HR, 3.07; 95% CI, 2.41-3.91) (*P* for interaction = .04) (Figure 3). In contrast, the interaction between p-tau217 and estrogen plus progestin was not significant for the combined MCI or dementia outcome or MCI (Figure 1 and Figure 2).

P-tau217 had larger magnitudes of association with MCI or dementia among White women (HR, 2.68; 95% CI, 2.38-3.01) compared with Black women (HR, 1.33; 95% CI, 1.04-1.70) (*P* for interaction < .001) (Figure 1), as well as dementia (Black women: HR, 1.90; 95% CI, 1.24-2.92; White women: HR, 3.42; 95% CI, 2.99-3.92; *P* for interaction = .01) (Figure 3). P-tau217 was not associated with MCI among Black women (HR, 1.19; 95% CI, 0.94-1.52) but was among White women (HR, 2.16; 95% CI, 1.90-2.47) (*P* for interaction < .001) (Figure 2). Associations of p-tau217 with the cognitive outcomes varied by *APOE* ε4, with larger magnitudes of association in carriers than noncarriers, an interaction that was significant for the combined MCI or dementia outcome (*P* for interaction = .02) (Figure 1) and, separately, for MCI (*P* for interaction = .02) (Figure 2), but not dementia when examined separately (*P* for interaction = .13) (Figure 3). There were also subgroup differences by age, with larger magnitudes of association for those aged older than 70 years vs 70 years or younger for MCI or dementia (*P* for interaction = .04) (Figure 1) and dementia (*P* for interaction = .04) (Figure 3) but not MCI (*P* for interaction = .53) (Figure 2).

**Figure 4.**
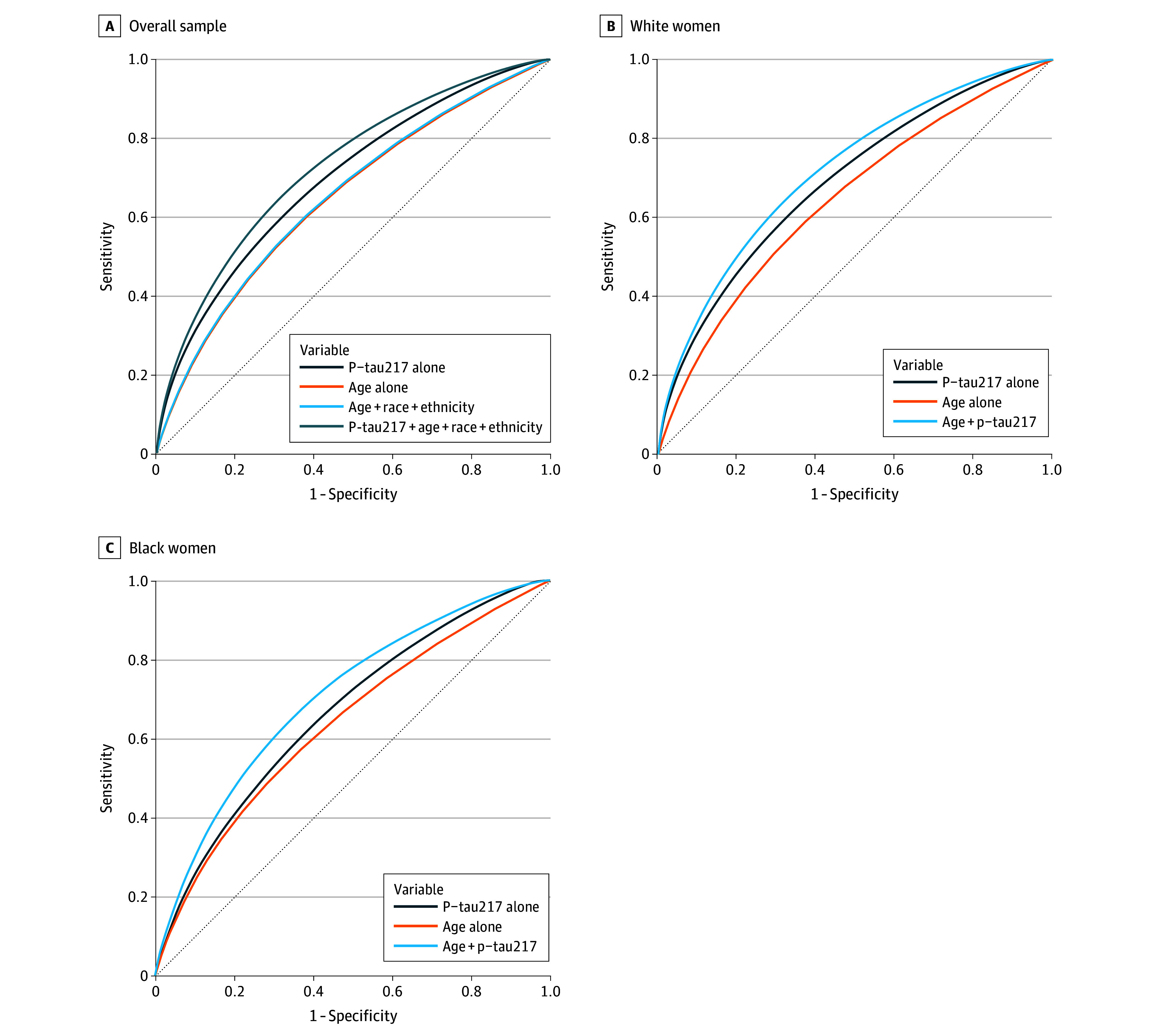
Discriminative Accuracy of Plasma Phosphorylated Tau 217 (p-tau217) for Incident Dementia in the Overall Sample, White Women, and Black Women Receiver operating characteristic curves were generated from Cox proportional hazards regression models that estimated discriminatory accuracy at the median follow-up of 15.0 years in the full sample, 16.4 years in White women, and 8.0 years in Black women.

P-tau217 showed better discrimination than demographics for dementia; the combination of p-tau217 and demographics outperformed either alone (area under the ROC [AUC] = 72.7%; 95% CI, 71.0%-74.6%) (Figure 4 and eTable 3 in Supplement 1). In race-stratified analyses of dementia, p-tau217 alone had slightly higher performance in White women compared with Black women (Figure 4 and eTable 3 in Supplement 1). The performance of the combination of p-tau217 and age was similar in White women (AUC = 72.0%; 95% CI, 70.3%-73.6%) and Black women (AUC = 70.4%; 95% CI, 64.0%-78.0%). AUCs for the combination of age and p-tau217 were higher in White women compared with Black women when examining MCI or dementia and MCI (eFigure 3 and eFigure 4 in Supplement 1).

In sensitivity analyses, we observed the largest magnitudes of association in the fourth quartile of p-tau217 across all outcomes (fourth vs first quartile for dementia: HR, 7.37; 95% CI, 5.30-10.25) (eTable 4 in Supplement 1). Findings were similar after excluding women with eGFR of 60 mL/min/1.73 m^2^ or less (eFigure 5 in Supplement 1). In Fine-Gray regression models, there were significant associations for all cognitive outcomes (eFigure 6 in Supplement 1). Cumulative incidence curves of MCI or dementia by p-tau217 are shown in eFigure 7 in Supplement 1. Women in the highest quartile of p-tau217 showed a higher incidence of MCI or dementia shortly after baseline, a pattern observed in both White and Black women. For women in the third quartile of p-tau217, higher incidence of dementia became apparent after approximately 15 years, with little increase in dementia incidence for those in the first and second quartiles over the 25-year follow-up.

## Discussion

In this cohort study among cognitively unimpaired older women, higher plasma p-tau217 was associated with incident MCI or dementia for up to 25 years of follow-up. We found that higher p-tau217 had a larger magnitude of association with incident dementia among women randomized to estrogen plus progestin compared with placebo, but associations did not vary by estrogen alone vs placebo. P-tau217 had a larger magnitude of association with MCI or dementia among White compared with Black women, although there was similar discriminative accuracy for dementia in both racial groups when combining p-tau217 with age.

In the original WHIMS trials, estrogen alone vs placebo was associated with a 38% increased risk of MCI or dementia (HR, 1.38; 95% CI, 1.01-1.89) but was not significantly associated with increased risk for each individual outcome,^23^ whereas estrogen plus progestin vs placebo was associated with double the risk of dementia (HR, 2.05; 95% CI, 1.21-3.48) but was not significantly associated with the risk of MCI (HR, 1.07; 95% CI, 0.74-1.55) or with the combined MCI or dementia outcome (HR, 1.37; 95% CI, 0.99-1.89).^22^ Here, we observed that higher baseline levels of p-tau217 were associated with greater risk of dementia among women randomized to estrogen plus progestin vs placebo; associations did not significantly vary by estrogen alone vs placebo. When examining MCI or dementia or MCI, separately, findings did not vary by treatment group for either HT regimen. It is important for future studies to determine the potential modifying role of HT on the association of p-tau217 with dementia.

There has been limited research on whether HT interacts with existing AD pathology to affect dementia risk. A study that included 193 cognitively unimpaired women found that HT users with higher amyloid-β positron emission tomography (PET) burden showed higher regional tau PET burden relative to nonusers.^17^ Further, late initiation of HT (>5 years after menopause) was associated with higher tau PET compared with early initiation (within 5 years of menopause).^17^ However, this was a cross-sectional study of self-reported HT use; thus, causality cannot be determined.

We observed a 3-fold higher hazard of all-cause dementia with higher baseline p-tau217 levels. A study among 198 community-dwelling, dementia-free participants reported that plasma p-tau217 was associated with over 2-fold higher hazard of AD over the 4-year follow-up.^10^ Among 435 cognitively unimpaired adults in the Biomarkers for Identifying Neurodegenerative Disorders Early and Reliably (BioFINDER) trial, plasma p-tau217 was associated with 2-fold higher hazard of all-cause dementia during an average of 4.8 years of follow-up.^7^ A study among participants with cognitive complaints found that plasma p-tau217 was associated with incident AD within 4 years, with an AUC of 0.83.^9^ We also found that the highest vs lowest quartile of p-tau217 was associated with 7-fold higher hazard of dementia. A study among 2148 dementia-free adults (including those with MCI) reported 3-fold higher hazard of all-cause dementia during the 16-year follow-up in those in the highest vs lowest quartile of baseline plasma p-tau217, with greater risk in women vs men.^8^ These findings support a potential dose-response association of p-tau217 with incident dementia, with markedly higher incidence at the highest quartile.

We found that the association of p-tau217 with MCI or dementia was greater among women older than 70 years and among *APOE* ε4 carriers. These findings are consistent with higher incident MCI or dementia when p-tau217 levels are high in the presence of the most potent dementia risk factors, age and *APOE* ε4. A recent study among 2148 dementia-free adults observed higher hazard of dementia for increasing plasma p-tau217 levels among *APOE* ε4 carriers vs noncarriers but reported that the association was larger in magnitude among those who were younger than 78 years vs 78 years or older; in contrast with our study, the sample included both men and women, as well as individuals with MCI.^8^

Although the long-term association of p-tau217 with MCI has not been studied as extensively as dementia, our results among White women are consistent with prior studies. A recent study among 1474 cognitively unimpaired adults reported that baseline plasma p-tau217 was associated with incident MCI during a median 3.8-year follow-up (HR, 1.57; 95% CI, 1.43-1.72).^31^ The Mayo Clinic Study of Aging and BioFINDER-2 cohorts reported similar findings.^32^

Both White and Black women showed higher hazard of dementia for higher levels of p-tau217, with a larger magnitude of the hazard ratio in White women. We also found that the combination of age and p-tau217 showed better ability to discriminate women who developed dementia from those who did not, with similar AUCs between White and Black women. However, there was no significant association of p-tau217 with incident MCI among Black women. None of the prior studies examined differences in p-tau217 associations with MCI by race. Although few studies have examined plasma p-tau217 associations with PET evidence of amyloid and tau pathology in Black adults, existing evidence suggests that plasma p-tau217 may be similarly sensitive to these brain pathologies in Black and White adults.^14,16^ The lack of association of p-tau217 with MCI among Black women in our study may indicate differences in MCI cause between racial groups (eg, vascular disease in Black women) or reflect misdiagnosis of MCI in Black women due to inequities in the accuracy of cognitive tests.^33,34^ It is also possible that racial differences in these associations arise from differences in demographics, comorbidities, and other characteristics between Black and White women that we could not fully control for and from the lower sample size in Black compared with White women.

### Limitations and Strengths

Our study has several limitations. We examined only older women. We did not examine AD because WHIMS did not classify dementia according to subtype; thus, AUC estimates were likely attenuated for all-cause dementia relative to AD-specific analyses. The sample size for Black women was smaller relative to that of White women, and residual confounding may have affected the results. The HT formulations in WHI may not be generalizable to currently favored HT regimens with lower doses, transdermal preparations, or micronized progesterone. Whether findings can be generalized to HT initiated earlier in menopause is unknown.

Study strengths include the large, diverse sample of community-dwelling women with extensive follow-up. The WHIMS HT trials had minimal exclusion criteria so that the study results would be maximally generalizable to postmenopausal women. Women were randomized to HT or placebo, providing the unique opportunity to assess whether associations of p-tau217 with MCI or dementia differed by HT regimen, without the usual challenges invoked by self-selection and self-report of HT use.

### Conclusions

In this cohort study, higher baseline plasma p-tau217 was associated with incident MCI or dementia for up to 25 years of follow-up among older women. Including p-tau217 improved discrimination of dementia relative to demographic characteristics alone, with similar performance in Black and White women. However, the performance of p-tau217 for MCI was lower in Black compared with White women, suggesting a need to further investigate MCI as a diagnostic construct across racial groups. P-tau217 had a larger magnitude of association with dementia among women assigned to estrogen plus progestin compared with placebo. P-tau217 associations with MCI or dementia were larger in magnitude for women older than 70 years and *APOE* ε4 carriers. Our findings suggest a potential moderating role of age, race, *APOE* ε4, and HT in the associations between p-tau217 and cognitive outcomes; further studies are needed to confirm these findings. Overall, our results support the value of plasma p-tau217 as an easily measured biomarker for future MCI or dementia that may have a variety of uses in both research and clinical practice among diverse populations.

## Notes

Supplement 1. eFigure 1. Study flow diagram eMethods. eTable 1. Baseline characteristics by inclusion in analytic sample, Women’s Health Initiative Memory Study, 1996-1999 eTable 2. Baseline characteristics by race, Women’s Health Initiative Memory Study, 1996-1999 eFigure 2. Violin plot of baseline plasma p-tau217 overall and by Black and White race eTable 3. Discriminative accuracy of plasma p-tau217 for dementia eFigure 3. Discriminative accuracy of plasma p-tau217 for the combined endpoint of mild cognitive impairment/dementia eFigure 4. Discriminative accuracy of plasma p-tau217 for mild cognitive impairment eTable 4. Associations of quartiles of baseline plasma p-tau217 levels with incident cognitive outcomes eFigure 5. Association of baseline plasma p-tau217 at baseline with incident cognitive outcomes, after excluding 524 women with eGFR ≤60 ml/min/1.73 m^2^ (N=2,170) eFigure 6. Association of baseline plasma p-tau217 with incident cognitive outcomes, accounting for competing risk of death using Fine-Gray models eFigure 7. Weighted cumulative incidence curves of the combined endpoint of MCI/dementia and separately dementia across quartiles of baseline plasma p-tau217

Supplement 2. Data Sharing Statement

## References

[1] Blennow K, Galasko D, Perneczky R, . The potential clinical value of plasma biomarkers in Alzheimer’s disease. Alzheimers Dement. 2023;19(12):5805-5816. doi:10.1002/alz.13455 37694991

[2] Janelidze S, Bali D, Ashton NJ, . Head-to-head comparison of 10 plasma phospho-tau assays in prodromal Alzheimer’s disease. Brain. 2023;146(4):1592-1601. doi:10.1093/brain/awac333 36087307 PMC10115176

[3] Schindler SE, Petersen KK, Saef B, ; Alzheimer’s Disease Neuroimaging Initiative (ADNI) Foundation for the National Institutes of Health (FNIH) Biomarkers Consortium Plasma Aβ and Phosphorylated Tau as Predictors of Amyloid and Tau Positivity in Alzheimer’s Disease Project Team. Head-to-head comparison of leading blood tests for Alzheimer’s disease pathology. Alzheimers Dement. 2024;20(11):8074-8096. doi:10.1002/alz.14315 39394841 PMC11567821

[4] Arranz J, Zhu N, Rubio-Guerra S, . Diagnostic performance of plasma pTau_217_, pTau_181_, Aβ_1-42_ and Aβ_1-40_ in the LUMIPULSE automated platform for the detection of Alzheimer disease. Alzheimers Res Ther. 2024;16(1):139. doi:10.1186/s13195-024-01513-9 38926773 PMC11200993

[5] Palmqvist S, Janelidze S, Quiroz YT, . Discriminative accuracy of plasma phospho-tau217 for Alzheimer disease vs other neurodegenerative disorders. JAMA. 2020;324(8):772-781. doi:10.1001/jama.2020.12134 32722745 PMC7388060

[6] Therriault J, Servaes S, Tissot C, . Equivalence of plasma p-tau217 with cerebrospinal fluid in the diagnosis of Alzheimer’s disease. Alzheimers Dement. 2023;19(11):4967-4977. doi:10.1002/alz.13026 37078495 PMC10587362

[7] Cullen NC, Leuzy A, Janelidze S, . Plasma biomarkers of Alzheimer’s disease improve prediction of cognitive decline in cognitively unimpaired elderly populations. Nat Commun. 2021;12(1):3555. doi:10.1038/s41467-021-23746-0 34117234 PMC8196018

[8] Grande G, Valletta M, Rizzuto D, . Blood-based biomarkers of Alzheimer’s disease and incident dementia in the community. Nat Med. 2025;31(6):2027-2035. doi:10.1038/s41591-025-03605-x 40140622 PMC12176656

[9] Palmqvist S, Tideman P, Cullen N, ; Alzheimer’s Disease Neuroimaging Initiative. Prediction of future Alzheimer’s disease dementia using plasma phospho-tau combined with other accessible measures. Nat Med. 2021;27(6):1034-1042. doi:10.1038/s41591-021-01348-z 34031605

[10] Yu L, Wang T, Hansson O, . MRI-derived AD signature of cortical thinning and plasma p-Tau217 for predicting Alzheimer dementia among community-dwelling older adults. Neurol Clin Pract. 2024;14(3):e200291. doi:10.1212/CPJ.0000000000200291 38720951 PMC11073883

[11] Brickman AM, Manly JJ, Honig LS, . Plasma p-tau181, p-tau217, and other blood-based Alzheimer’s disease biomarkers in a multi-ethnic, community study. Alzheimers Dement. 2021;17(8):1353-1364. doi:10.1002/alz.12301 33580742 PMC8451860

[12] Petersen ME, Zhang F, Hall J, ; HABS-HD Study Team. Characterization of plasma AT(N) biomarkers among a racial and ethnically diverse community-based cohort: an HABS-HD study. Alzheimers Dement (N Y). 2025;11(1):e70045. doi:10.1002/trc2.70045 39975470 PMC11837735

[13] Molina-Henry DP, Raman R, Liu A, ; AHEAD 3-45 Study Team. Racial and ethnic differences in plasma p-tau217 ratio biomarker eligibility rates in a preclinical AD trial with lecanemab. Alzheimers Dement (Amst). 2025;17(3):e70164. doi:10.1002/dad2.70164 40861822 PMC12371446

[14] Cousins KAQ, Korecka M, Wan Y, ; and the Alzheimer’s Disease Neuroimaging Initiative. Comparison of plasma p-tau217/Aβ42, p-tau217, and Aβ42/Aβ40 biomarkers by race to detect Alzheimer’s disease. Alzheimers Dement. 2025;21(8):e70469. doi:10.1002/alz.70469 40801297 PMC12344574

[15] Rudolph MD, Sutphen CL, Register TC, . Evaluation of plasma p-tau217 for detecting amyloid pathology in a heterogeneous community-based cohort. Alzheimers Dement. 2025;21(7):e70426. doi:10.1002/alz.70426 40613474 PMC12231229

[16] Ennis GE, Norton D, Langhough RE, . The performance of plasma p-tau217 in Black middle-aged and older adults. Alzheimers Dement. 2025;21(5):e70288. doi:10.1002/alz.70288 40410928 PMC12101965

[17] Coughlan GT, Betthauser TJ, Boyle R, . Association of age at menopause and hormone therapy use with tau and β-amyloid positron emission tomography. JAMA Neurol. 2023;80(5):462-473. doi:10.1001/jamaneurol.2023.0455 37010830 PMC10071399

[18] Coughlan GT, Rubinstein Z, Klinger H, . Associations between hormone therapy use and tau accumulation in brain regions vulnerable to Alzheimer’s disease. Sci Adv. 2025;11(10):eadt1288. doi:10.1126/sciadv.adt1288 40043125 PMC11881894

[19] Jauregi-Zinkunegi A, Gleason CE, Bendlin B, . Menopausal hormone therapy is associated with worse levels of Alzheimer’s disease biomarkers in APOE ε4-carrying women: an observational study. Alzheimers Dement. 2025;21(2):e14456. doi:10.1002/alz.14456 39783876 PMC11848176

[20] Pourhadi N, Mørch LS, Holm EA, Torp-Pedersen C, Meaidi A. Menopausal hormone therapy and dementia: nationwide, nested case-control study. BMJ. 2023;381:e072770. doi:10.1136/bmj-2022-072770 37380194 PMC10302215

[21] Pourhadi N, Mørch LS, Holm EA, Torp-Pedersen C, Meaidi A. Dementia in women using estrogen-only therapy. JAMA. 2024;331(2):160-162. doi:10.1001/jama.2023.23784 38109125 PMC10728800

[22] Shumaker SA, Legault C, Rapp SR, ; WHIMS Investigators. Estrogen plus progestin and the incidence of dementia and mild cognitive impairment in postmenopausal women: the Women’s Health Initiative Memory Study: a randomized controlled trial. JAMA. 2003;289(20):2651-2662. doi:10.1001/jama.289.20.2651 12771112

[23] Shumaker SA, Legault C, Kuller L, ; Women’s Health Initiative Memory Study. Conjugated equine estrogens and incidence of probable dementia and mild cognitive impairment in postmenopausal women: women’s health initiative memory study. JAMA. 2004;291(24):2947-2958. doi:10.1001/jama.291.24.2947 15213206

[24] Espeland MA, Rapp SR, Manson JE, ; WHIMSY and WHIMS-ECHO Study Groups. Long-term effects on cognitive trajectories of postmenopausal hormone therapy in two age groups. J Gerontol A Biol Sci Med Sci. 2017;72(6):838-845. doi:10.1093/gerona/glw156 27506836 PMC6075542

[25] Petersen RC, Doody R, Kurz A, . Current concepts in mild cognitive impairment. Arch Neurol. 2001;58(12):1985-1992. doi:10.1001/archneur.58.12.1985 11735772

[26] American Psychiatric Association. Diagnostic and Statistical Manual of Mental Disorders. 4th ed. American Psychiatric Association; 1994.

[27] Inker LA, Eneanya ND, Coresh J, ; Chronic Kidney Disease Epidemiology Collaboration. New creatinine- and cystatin C–based equations to estimate GFR without race. N Engl J Med. 2021;385(19):1737-1749. doi:10.1056/NEJMoa2102953 34554658 PMC8822996

[28] Lu Y, Pike JR, Chen J, . Changes in Alzheimer disease blood biomarkers and associations with incident all-cause dementia. JAMA. 2024;332(15):1258-1269. doi:10.1001/jama.2024.6619 39068543 PMC11284635

[29] Gupta A, Mielke MM, Tariot PN. Detection of Alzheimer’s disease neuropathology in chronic kidney disease: current state and future directions. J Am Geriatr Soc. 2025;73(10):2995-3001. doi:10.1111/jgs.19554 40525987 PMC12354189

[30] Mielke MM, Fowler NR. Alzheimer disease blood biomarkers: considerations for population-level use. Nat Rev Neurol. 2024;20(8):495-504. doi:10.1038/s41582-024-00989-1 38862788 PMC11347965

[31] Ossenkoppele R, Salvadó G, Janelidze S, ; PREVENT-AD Research Group. Plasma p-tau217 and tau-PET predict future cognitive decline among cognitively unimpaired individuals: implications for clinical trials. Nat Aging. 2025;5(5):883-896. doi:10.1038/s43587-025-00835-z 40155777 PMC12092243

[32] Cogswell PM, Wiste HJ, Therneau TM, . Association of plasma Alzheimer’s disease biomarkers with cognitive decline in cognitively unimpaired individuals. Alzheimers Dement. 2025;21(9):e70625. doi:10.1002/alz.70625 40883967 PMC12397066

[33] Kiselica AM, Johnson E, Lewis KR, Trout K. Examining racial disparities in the diagnosis of mild cognitive impairment. Appl Neuropsychol Adult. 2023;30(6):749-756. doi:10.1080/23279095.2021.1976778 34554020 PMC8940745

[34] Raman R, Quiroz YT, Langford O, . Disparities by race and ethnicity among adults recruited for a preclinical Alzheimer disease trial. JAMA Netw Open. 2021;4(7):e2114364. doi:10.1001/jamanetworkopen.2021.14364 34228129 PMC8261604

