# Supplementary material for: Plasma Phosphorylated Tau 217 and Incident Mild Cognitive Impairment and Dementia in Older Women
Source: JAMA Netw Open. 2026 Mar 10;9(3):e261295. doi: 10.1001/jamanetworkopen.2026.1295 (PMC12976794; doi:10.1001/jamanetworkopen.2026.1295)
Supplement: Supplement 1. — eFigure 1. Study flow diagram eMethods. eTable 1. Baseline characteristics by inclusion in analytic sample, Women’s Health Initiative Memory Study, 1996-1999 eTable 2. Baseline characteristics by race, Women’s Health Initiative Memory Study, 1996-1999 eFigure 2. Violin plot of baseline plasma p-tau217 overall and by Black and White race eTable 3. Discriminative accuracy of plasma p-tau217 for dementia eFigure 3. Discriminative accuracy of plasma p-tau217 for the combined endpoint of mild cognitive impairment/dementia eFigure 4. Discriminative accuracy of plasma p-tau217 for mild cognitive impairment eTable 4. Associations of quartiles of baseline plasma p-tau217 levels with incident cognitive outcomes eFigure 5. Association of baseline plasma p-tau217 at baseline with incident cognitive outcomes, after excluding 524 women with eGFR ≤60 ml/min/1.73 m2 (N=2,170) eFigure 6. Association of baseline plasma p-tau217 with incident cognitive outcomes, accounting for competing risk of death using Fine-Gray models eFigure 7. Weighted cumulative incidence curves of the combined endpoint of MCI/dementia and separately dementia across quartiles of baseline plasma p-tau217 [file jamanetwopen-e261295-s001.pdf]

# Supplemental Online Content

Shadyab AH, Zhang B, LaCroix AZ, et al. Plasma phosphorylated tau 217 and incident mild cognitive impairment and dementia in older women. *JAMA Netw. Open*. 2026;9(3):e261295. doi:10.1001/jamanetworkopen.2026.1295

**eFigure 1.** Study flow diagram

**eMethods.**

**eTable 1.** Baseline characteristics by inclusion in analytic sample, Women's Health Initiative Memory Study, 1996-1999

**eTable 2.** Baseline characteristics by race, Women's Health Initiative Memory Study, 1996-1999

**eFigure 2.** Violin plot of baseline plasma p-tau217 overall and by Black and White race

**eTable 3.** Discriminative accuracy of plasma p-tau217 for dementia

**eFigure 3.** Discriminative accuracy of plasma p-tau217 for the combined endpoint of mild cognitive impairment/dementia

**eFigure 4.** Discriminative accuracy of plasma p-tau217 for mild cognitive impairment

**eTable 4.** Associations of quartiles of baseline plasma p-tau217 levels with incident cognitive outcomes

**eFigure 5.** Association of baseline plasma p-tau217 at baseline with incident cognitive outcomes, after excluding 524 women with eGFR ≤60 ml/min/1.73 m² (N=2,170)

**eFigure 6.** Association of baseline plasma p-tau217 with incident cognitive outcomes, accounting for competing risk of death using Fine-Gray models

**eFigure 7.** Weighted cumulative incidence curves of the combined endpoint of MCI/dementia and separately dementia across quartiles of baseline plasma p-tau217

This supplemental material has been provided by the authors to give readers additional information about their work.

© 2026 Shadyab AH et al. *JAMA Network Open*.

**eFigure 1. Study flow diagram**

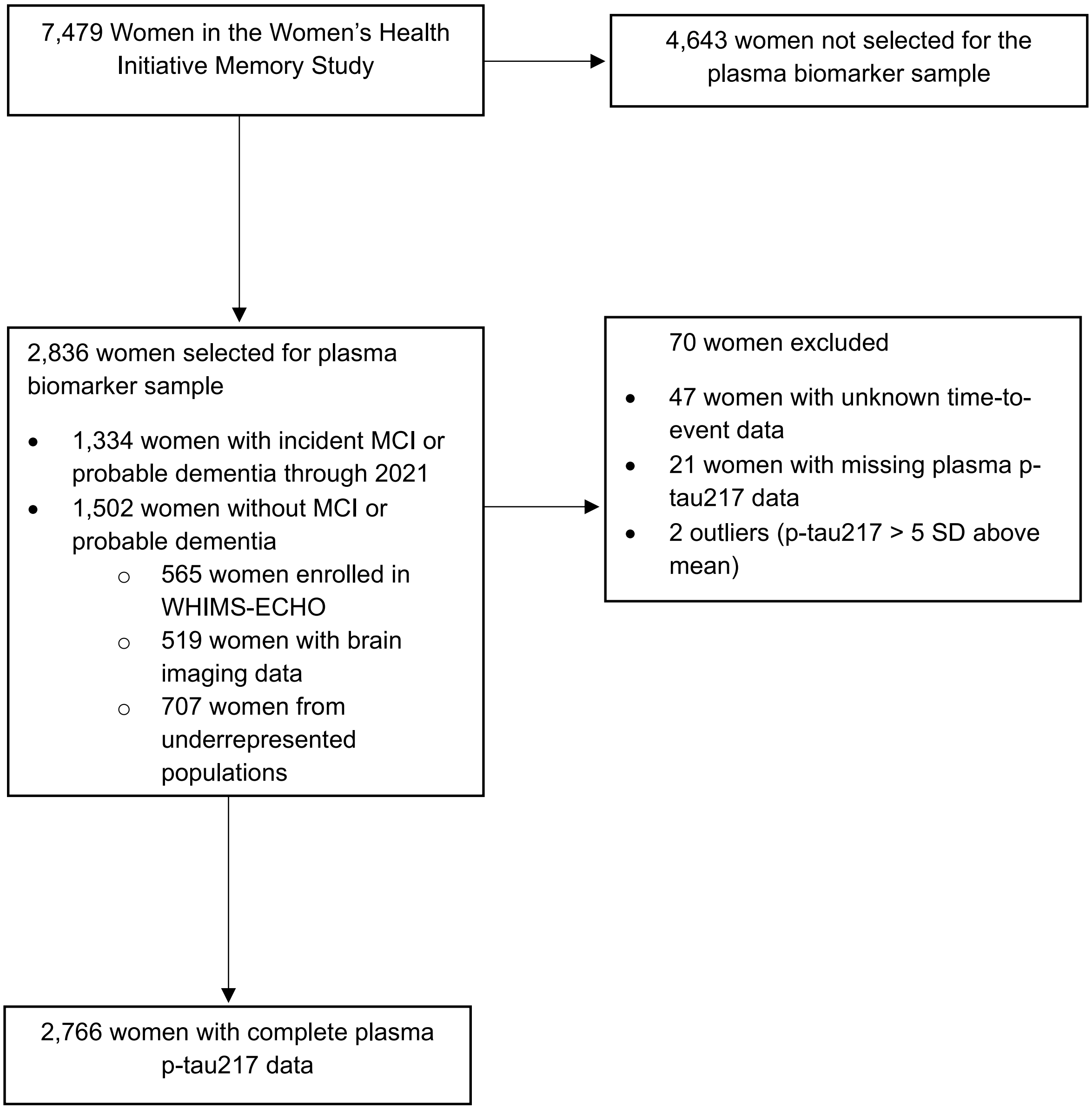


© 2026 Shadyab AH et al. *JAMA Network Open*.

## eMethods

### Sample Selection

Among the 7,479 WHIMS participants, we first selected all 1,334 women with incident MCI or probable dementia through the end of follow-up on November 3, 2021. We next selected 1,502 controls who did not have MCI or probable dementia during follow-up, including 565 who were enrolled in WHIMS-ECHO. Controls in our sample included all women who had brain imaging data (n=519) and all women from underrepresented populations (including American Indian/Alaskan Native, Asian, Native Hawaiian/Other Pacific Islander, Black, more than one race, and Hispanic/Latina; n=707). We excluded participants with unknown time-to-event data (N=47), missing plasma p-tau217 data (N = 21), and two outliers more than five standard deviations above the mean for p-tau217, leading to a final analytic sample of 2,766 women. The Cox models were focused on women without missing race or ethnicity (N=2,694), as these variables were not imputed in the analysis.

### Plasma P-tau217 Measurement

The following interassay laboratory coefficients of variation (CVs) were derived from an ARDL pooled sample and the two kit controls, which were run on every plate along with the samples: 11.4%, 11.2%, and 12.9% at mean concentrations of 0.75, 0.39, and 0.15 pg/mL for plasma p-tau217, respectively. The lower limit of detection (LOD) was 0.012 pg/mL, and the lower limit of quantification was 0.06 pg/mL. No values were less than the LOD in our analytic sample.

### MCI and Dementia Adjudication

Briefly, participants completed the Modified Mini-Mental State Examination, with those scoring below specific cut points (<80 for those with <8 years of education and <88 for those with ≥9 years of education) completing a modified Consortium to Establish a Registry for AD battery of neuropsychological tests and standardized tests in person. A physician with expertise in dementia diagnosis classified women as having no dementia, MCI, or probable dementia. MCI diagnosis was based on Petersen's criteria, and dementia diagnosis was based on *Diagnostic and Statistical Manual of Mental Disorders, Fourth Edition (DSM-IV)* criteria.[1,2] All data were sent to the WHIMS Clinical Coordinating Center for review and central adjudication of final diagnosis by a panel consisting of a neurologist, geriatric psychiatrist, and geropsychologist. WHIMS-ECHO used a common, validated protocol of telephone-based cognitive assessments and informant reviews, and a similar protocol to that of WHIMS for ascertainment and central adjudication of final diagnosis.[3]

### Calculation of Weights

To account for differences between the full WHIMS cohort and the biomarker analytic sample, we generated inverse probability weights (IPW) for selection into the biomarker sample. Propensity scores for IPW were estimated using logistic regression in the full WHIMS cohort (N=7,479) based on covariates that may influence selection into the sample. The model estimated the probability of inclusion in the biomarker sample as a function of age, region, race, ethnicity, baseline smoking status, hormone therapy trial arm, baseline cardiovascular disease, diabetes, cancer, depressive symptoms, hysterectomy, prior hormone use, body mass index, hypertension, MCI/dementia diagnosis, and participation in Women's Health Initiative (WHI) ancillary studies (i.e., brain imaging and the Long Life Study).

To account for oversampling of MCI/dementia cases in our analytic sample, we generated sampling weights so that the weighted analytic sample reflected the population incidence of MCI/probable dementia in WHIMS.[1,2] Final weights for analysis were calculated as the product of IPW and the sampling weights. All analyses incorporated these final weights to generate estimates that were generalizable to the full WHIMS cohort.

### References

1. Petersen RC, Doody R, Kurz A, et al. Current concepts in mild cognitive impairment. Arch Neurol 2001;58(12):1985-92, doi:10.1001/archneur.58.12.1985
2. American Psychiatric Association. Diagnostic and Statistical Manual of Mental Disorders, Fourth Edition. Washington DC: American Psychiatric Association: 1994.

3. Espeland MA, Rapp SR, Manson JE, et al. Long-term Effects on Cognitive Trajectories of Postmenopausal Hormone Therapy in Two Age Groups. J Gerontol A Biol Sci Med Sci 2017;72(6):838-845, doi:10.1093/gerona/glw156
4. Rose S, van der Laan MJ. A Note on Risk Prediction for Case-Control Studies. *U.C. Berkeley Division of Biostatistics Working Paper Series.* 2008.
5. van der Laan MJ. Estimation based on case-control designs with known prevalence probability. Int J Biostat 2008;4(1):Article 17, doi:10.2202/1557-4679.1114

© 2026 Shadyab AH et al. *JAMA Network Open*.

**eTable 1. Baseline characteristics by inclusion in analytic sample, Women's Health Initiative Memory Study, 1996-1999**

| | Analytic Sample (N = 2,766) | Not in Analytic Sample (N = 4,713) | Full WHIMS (N = 7,479) | p-value |
|---|---|---|---|---|
| **Age, Mean (SD)** | 69.9 (3.8) | 70.3 (3.9) | 70.1 (3.8) | <.001 |
| **Hormone therapy treatment arm, n (%)** | | | | .05 |
| Estrogen-alone placebo | 569 (20.6%) | 915 (19.4%) | 1,484 (19.8%) | |
| Estrogen-alone intervention | 570 (20.6%) | 899 (19.1%) | 1,469 (19.6%) | |
| Estrogen plus progestin placebo | 803 (29.0%) | 1,499 (31.8%) | 2,302 (30.8%) | |
| Estrogen plus progestin intervention | 824 (29.8%) | 1,400 (29.7%) | 2,224 (29.7%) | |
| **Race, n (%)** | | | | <.001 |
| American Indian or Alaskan Native | 17 (0.6%) | 0 (0.0%) | 17 (0.2%) | |
| Asian | 121 (4.5%) | 6 (0.1%) | 127 (1.7%) | |
| Black | 486 (17.9%) | 37 (0.8%) | 523 (7.1%) | |
| More than one race | 75 (2.8%) | 5 (0.1%) | 80 (1.1%) | |
| Native Hawaiian or other Pacific Islander | 8 (0.3%) | 0 (0.0%) | 8 (0.1%) | |
| White | 2,007 (73.9%) | 4,642 (99.0%) | 6,649 (89.8%) | |
| Unknown/not reported | 52 | 23 | 75 | |
| **Ethnicity, n (%)** | | | | <.001 |
| Hispanic or Latino | 196 (7.1%) | 18 (0.4%) | 214 (2.9%) | |
| Not Hispanic or Latino | 2,548 (92.9%) | 4,685 (99.6%) | 7,233 (97.1%) | |
| Unknown/not reported | 22 | 10 | 32 | |
| **BMI, Mean (SD)** | 28.6 (5.6) | 28.5 (5.7) | 28.5 (5.7) | .29 |
| Missing | 13 | 30 | 43 | |
| **Smoking Status, n (%)** | | | | <.001 |
| Never Smoked | 1,556 (57.1%) | 2,353 (50.7%) | 3,909 (53.1%) | |
| Past Smoker | 1,024 (37.6%) | 1,906 (41.1%) | 2,930 (39.8%) | |
| Current Smoker | 146 (5.4%) | 383 (8.3%) | 529 (7.2%) | |
| Missing | 40 | 71 | 111 | |
| **Education, n (%)** | | | | <.001 |
| Less than high school equivalent | 256 (9.3%) | 320 (6.8%) | 576 (7.7%) | |
| High school diploma or GED | 609 (22.1%) | 1,038 (22.1%) | 1,647 (22.1%) | |
| Vocational, training school, or some college or associate | 1,031 (37.4%) | 1,971 (41.9%) | 3,002 (40.3%) | |
| College graduate or higher | 861 (31.2%) | 1,371 (29.2%) | 2,232 (29.9%) | |
| Missing | 9 | 13 | 22 | |
| **Diabetes, n (%)** | 190 (6.9%) | 298 (6.3%) | 488 (6.5%) | .35 |
| Missing | 6 | 8 | 14 | |
| **Cardiovascular disease, n (%)** | 134 (4.8%) | 232 (4.9%) | 366 (4.9%) | .88 |

© 2026 Shadyab AH et al. *JAMA Network Open*.

| | Analytic Sample (N = 2,766) | Not in Analytic Sample (N = 4,713) | Full WHIMS (N = 7,479) | p-value |
|---|---|---|---|---|
| **Physical activity (hours/week), Mean (SD)** | 11.5 (13.7) | 11.1 (13.0) | 11.3 (13.3) | .31 |
| Missing | 8 | 9 | 17 | |
| **Total cholesterol (mg/dL), Mean (SD)** | 234.2 (39.5) | 234.5 (40.3) | 234.4 (40.0) | .99 |
| Missing | 429 | 556 | 985 | |
| **HDL cholesterol (mg/dL), Mean (SD)** | 53.6 (12.4) | 53.3 (12.6) | 53.4 (12.6) | .28 |
| Missing | 429 | 556 | 985 | |
| **Hypertension, n (%)** | 1,948 (70.7%) | 3,321 (70.6%) | 5,269 (70.6%) | .96 |
| Missing | 10 | 11 | 21 | |
| **eGFR (ml/min/1.73 m²), Mean (SD)** | 83.7 (13.5) | 84.2 (13.5) | 84.0 (13.5) | .07 |
| Missing | 430 | 556 | 986 | |
| ***APOE* ε4 carrier status, n (%)** | | | | .07 |
| No ε4 alleles | 1,330 (73.2%) | 3,053 (75.4%) | 4,383 (74.8%) | |
| At least one ε4 allele | 486 (26.8%) | 994 (24.6%) | 1,480 (25.2%) | |
| Missing | 950 | 666 | 1,616 | |

BMI, body mass index; CVD, cardiovascular disease; E-alone, estrogen-alone therapy; E+P, estrogen plus progestin therapy; eGFR, estimated glomerular filtration rate; HDL, high-density lipoprotein; mg/dL, milligrams per deciliter; ml/min/1.73 m², milliliters per minute per 1.73 square meters; SD, standard deviation

© 2026 Shadyab AH et al. *JAMA Network Open*.

**eTable 2. Baseline characteristics by race, Women's Health Initiative Memory Study, 1996-1999**

| | Black<br>N = 486 | White<br>N = 2,007 | p-value |
|---|---|---|---|
| **Age, Mean (SD)** | 69.5 (3.7) | 69.9 (3.8) | .03 |
| **Hormone therapy treatment arm, n (%)** | | | <.001 |
| E-alone placebo | 139 (28.6%) | 374 (18.6%) | |
| E-alone intervention | 149 (30.7%) | 361 (18.0%) | |
| E+P placebo | 99 (20.4%) | 620 (30.9%) | |
| E+P intervention | 99 (20.4%) | 652 (32.5%) | |
| **Ethnicity, n (%)** | | | <.001 |
| Hispanic or Latino | 4 (0.8%) | 135 (6.7%) | |
| Not Hispanic or Latino | 482 (99.2%) | 1,872 (93.3%) | |
| **BMI, Mean (SD)** | 30.7 (6.1) | 28.2 (5.4) | <.001 |
| Missing | 4 | 8 | |
| **Smoking Status, n (%)** | | | <.001 |
| Never smoked | 240 (50.7%) | 1,153 (58.1%) | |
| Past smoker | 192 (40.6%) | 745 (37.6%) | |
| Current smoker | 41 (8.7%) | 86 (4.3%) | |
| Missing | 13 | 23 | |
| **Education, n (%)** | | | <.001 |
| Less than high school equivalent | 83 (17.2%) | 134 (6.7%) | |
| High school diploma or GED | 78 (16.1%) | 464 (23.2%) | |
| Some college or associate | 173 (35.8%) | 754 (37.7%) | |
| College graduate or higher | 149 (30.8%) | 649 (32.4%) | |
| Missing | 3 | 6 | |
| **Diabetes, n (%)** | 73 (15.1%) | 85 (4.2%) | <.001 |
| Missing | 2 | 4 | |
| **CVD, n (%)** | 39 (8.0%) | 76 (3.8%) | <.001 |
| **Physical activity (MET-hours/week), Mean (SD)** | 9.2 (13.3) | 12.0 (13.8) | <.001 |
| Missing | 2 | 5 | |
| **Total cholesterol (mg/dL), Mean (SD)** | 228.2 (45.3) | 235.4 (38.3) | <.001 |
| Missing | 126 | 103 | |
| **HDL cholesterol (mg/dL), Mean (SD)** | 55.7 (13.9) | 53.4 (12.2) | .004 |
| Missing | 126 | 103 | |
| **eGFR (ml/min/1.73 m²), Mean (SD)** | 77.2 (16.3) | 84.8 (12.5) | <.001 |
| Missing | 126 | 104 | |
| **Hypertension, n (%)** | 408 (84.3%) | 1,339 (67.0%) | <.001 |
| Missing | 2 | 7 | |

BMI, body mass index; CVD, cardiovascular disease; E-alone, estrogen-alone therapy; E+P, estrogen plus progestin therapy; eGFR, estimated glomerular filtration rate; HDL, high-density lipoprotein; mg/dL, milligrams per deciliter; ml/min/1.73 m², milliliters per minute per 1.73 square meters; SD, standard deviation

© 2026 Shadyab AH et al. *JAMA Network Open*.

**eFigure 2. Violin plot of baseline plasma p-tau217 overall and by Black and White race.** SD, standard deviation.

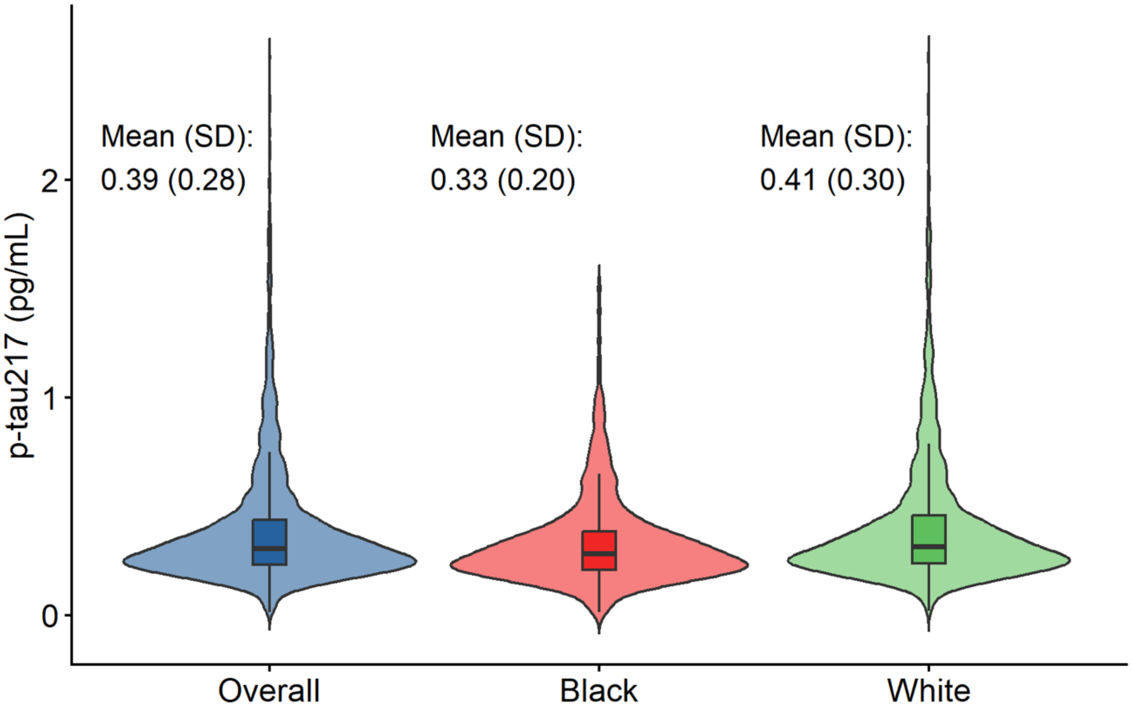


© 2026 Shadyab AH et al. *JAMA Network Open*.

**eTable 3. Discriminative accuracy of plasma p-tau217 for dementia**

| Predictor | AUC (95% CI) |
|---|---|
| **Total Sample** | |
| P-tau217 Alone | 69.6% (67.8%, 71.3%) |
| Age Alone | 64.7% (62.8%, 66.6%) |
| Age + Race + Ethnicity | 65.1% (63.1%, 67.0%) |
| P-tau217 + Age + Race + Ethnicity | 72.7% (71.0%, 74.6%) |
| **White Women** | |
| P-tau217 Alone | 69.3% (67.5%, 71.0%) |
| Age Alone | 64.6% (62.6%, 66.6%) |
| P-tau217 + Age | 72.0% (70.3%, 73.6%) |
| **Black Women** | |
| P-tau217 Alone | 66.4% (59.3%, 74.1%) |
| Age Alone | 63.7% (55.4%, 71.3%) |
| P-tau217 + Age | 70.4% (64.0%, 78.0%) |

AUC, area under the curve; CI, confidence interval

© 2026 Shadyab AH et al. *JAMA Network Open*.

**eFigure 3. Discriminative accuracy of plasma p-tau217 for the combined endpoint of mild cognitive impairment/dementia.** (a) p-tau217 alone and in combination with age, race, and ethnicity in the overall sample; (b) p-tau217 among White women; and (c) p-tau217 among Black women. Receiver operating characteristic curves were generated from Cox proportional hazards regression models that estimated discriminatory accuracy at the median follow-up of 14.1 years in the full sample, 15.4 years in White women, and 7.1 years in Black women. The sample included 2,694 women after removing those with missing race.

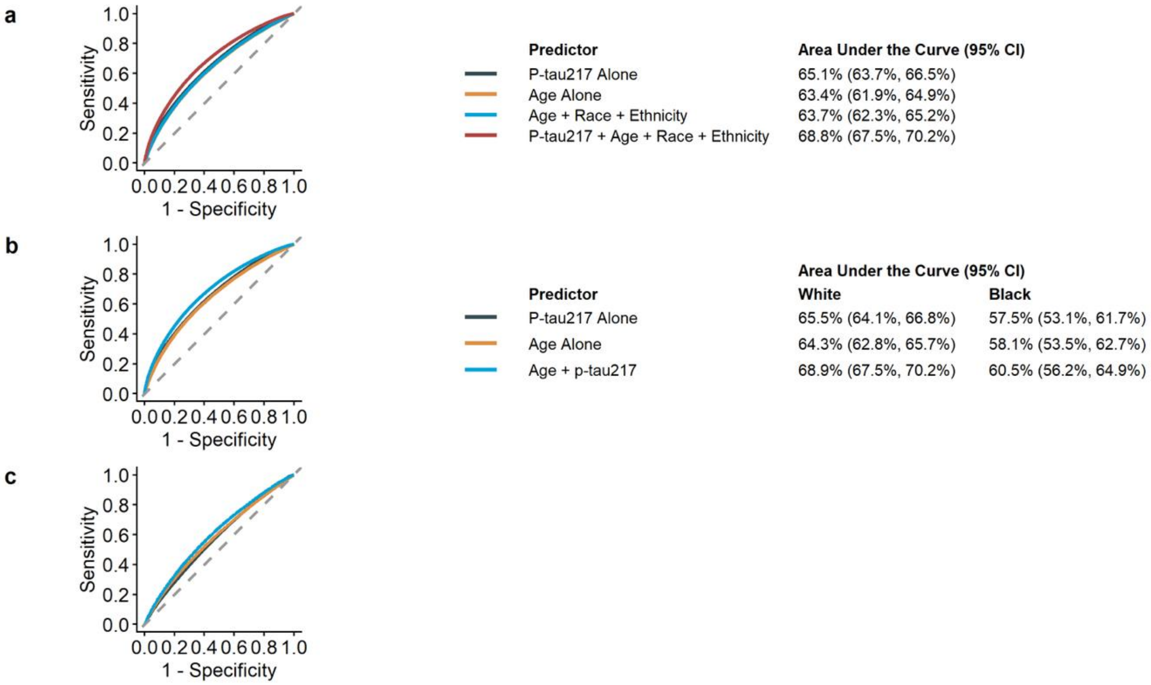


© 2026 Shadyab AH et al. *JAMA Network Open*.

**eFigure 4. Discriminative accuracy of plasma p-tau217 for mild cognitive impairment.** (a) p-tau217 alone and in combination with age, race, and ethnicity in the overall sample; (b) p-tau217 among White women; and (c) p-tau217 among Black women. Receiver operating characteristic curves were generated from Cox proportional hazards regression models that estimated discriminatory accuracy at the median follow-up of 14.1 years in the full sample, 15.7 years in White women, and 7.1 years in Black women. The sample included 2,694 women after removing those with missing race.

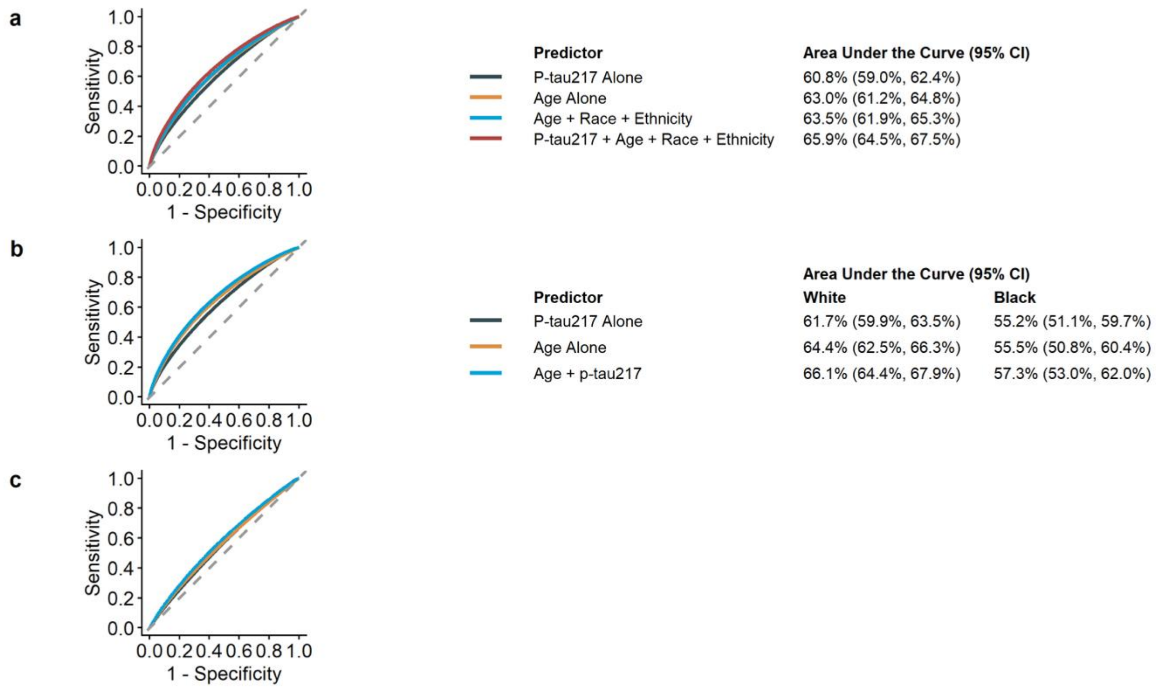


© 2026 Shadyab AH et al. *JAMA Network Open*.

## eTable 4. Associations of quartiles of baseline plasma p-tau217 levels with incident cognitive outcomes

| | Plasma P-tau217, pg/ml | HR (95% CI) |
|---|---|---|
| MCI/Dementia | 1st quartile: [0.014, 0.230) | 1 (reference) |
| | 2nd quartile: [0.230, 0.305) | 0.95 (0.73 - 1.25) |
| | 3rd quartile: [0.305, 0.438) | 1.44 (1.10 - 1.89) |
| | 4th quartile: [0.438, 2.564] | 4.69 (3.55 - 6.21) |
| MCI | 1st quartile: [0.014, 0.230) | 1 (reference) |
| | 2nd quartile: [0.230, 0.305) | 0.88 (0.66 - 1.17) |
| | 3rd quartile: [0.305, 0.438) | 1.36 (1.02 - 1.80) |
| | 4th quartile: [0.438, 2.564] | 3.30 (2.45 - 4.44) |
| Dementia | 1st quartile: [0.014, 0.230) | 1 (reference) |
| | 2nd quartile: [0.230, 0.305) | 1.06 (0.76 - 1.48) |
| | 3rd quartile: [0.305, 0.438) | 1.72 (1.24 - 2.37) |
| | 4th quartile: [0.438, 2.564] | 7.37 (5.30 - 10.25) |

CI, confidence interval; HR, hazard ratio; MCI, mild cognitive impairment

All models were adjusted for hormone therapy trial arm, age, race, ethnicity, education, body mass index, smoking status, diabetes, cardiovascular disease, hypertension, physical activity, estimated glomerular filtration rate, total cholesterol, and high-density lipoprotein cholesterol. Models were also weighted to account for sample selection.

© 2026 Shadyab AH et al. *JAMA Network Open*.

**eFigure 5. Association of baseline plasma p-tau217 at baseline with incident cognitive outcomes, *after excluding 524 women with eGFR ≤60 ml/min/1.73 $m^2$* (N=2,170).** Hazard ratios (HRs) and 95% confidence intervals (CI) were derived from Cox proportional hazards regression models. All models were adjusted for hormone therapy trial arm, age, race, ethnicity, education, body mass index, smoking status, diabetes, cardiovascular disease, hypertension, physical activity, estimated glomerular filtration rate, total cholesterol, and high-density lipoprotein cholesterol. Models were also weighted to account for sample selection. The number of events/at risk for the unweighted sample are shown.

| Outcome | Number of events/at risk | HR (95% CI) |
|---|---|---|
| MCI/Probable Dementia | 1105/2170 | 2.46 (2.18, 2.78) |
| MCI | 700/2170 | 1.93 (1.68, 2.22) |
| Probable Dementia | 654/2170 | 3.22 (2.80, 3.70) |

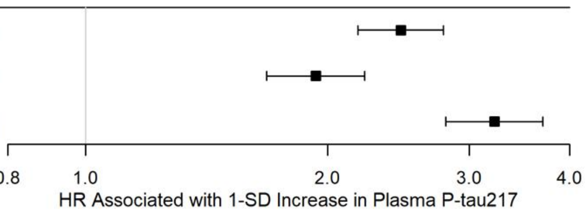

**eFigure 6. Association of baseline plasma p-tau217 with incident cognitive outcomes, *accounting for competing risk of death using Fine-Gray models*.** Subdistribution hazard ratios (SHRs) and 95% confidence intervals (CI) are shown. All models were adjusted for hormone therapy trial arm, age, race, ethnicity, education, body mass index, smoking status, diabetes, cardiovascular disease, hypertension, physical activity, estimated glomerular filtration rate, total cholesterol, and high-density lipoprotein cholesterol. Models were also weighted to account for sample selection. The sample included 2,694 women after removing those with missing race. MCI, mild cognitive impairment.

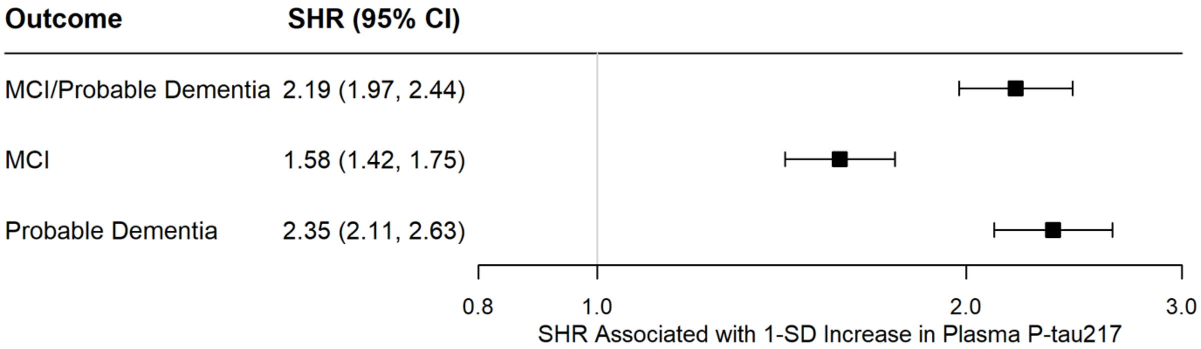


© 2026 Shadyab AH et al. *JAMA Network Open*.

**eFigure 7. Weighted cumulative incidence curves of the combined endpoint of MCI/dementia and separately dementia across quartiles of baseline plasma p-tau217.** Follow-up was capped at the 90th percentile due to smaller risk sets from censoring leading to unstable survival estimates after 20 years of follow-up. Numbers at risk are not shown, as cumulative incidence curves were weighted using a pseudopopulation.

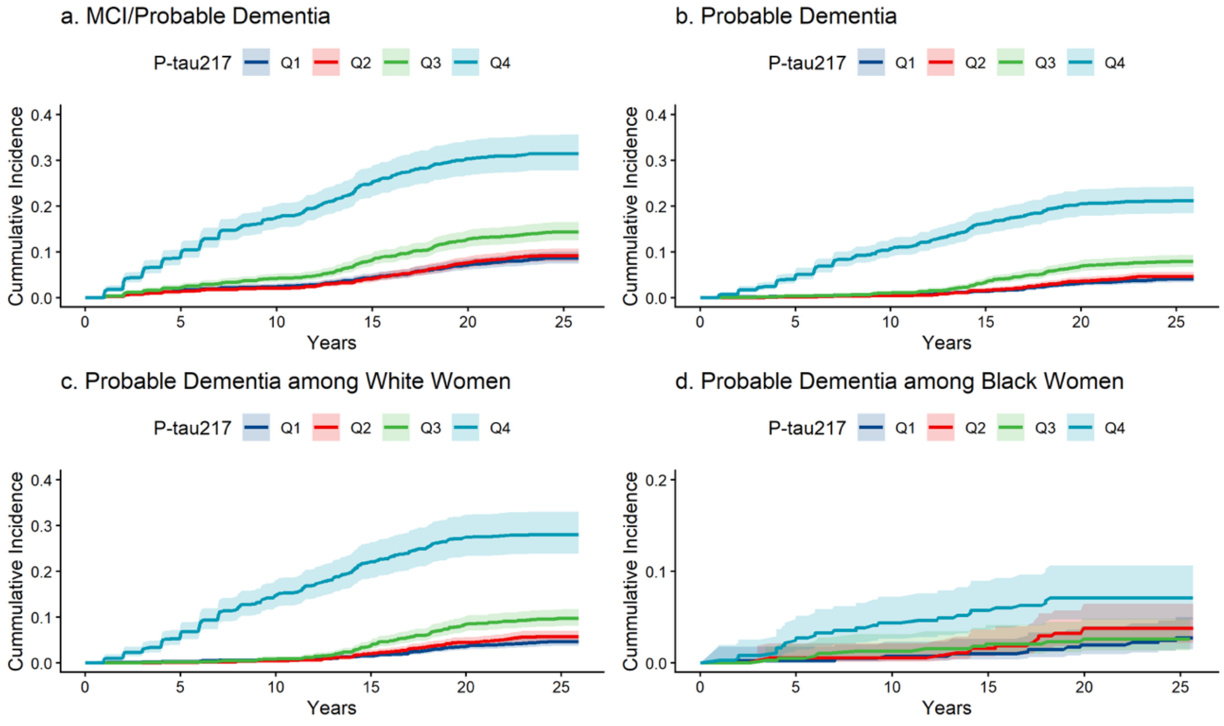


© 2026 Shadyab AH et al. *JAMA Network Open*.
