# Supplementary material for: Plasma Phosphorylated Tau 217 and Incident Mild Cognitive Impairment and Dementia in Older Women
Source: JAMA Netw Open. 2026 Mar 10;9(3):e261295. doi: 10.1001/jamanetworkopen.2026.1295 (PMC12976794; doi:10.1001/jamanetworkopen.2026.1295)
Supplement: Supplement 2. — Data Sharing Statement [file jamanetwopen-e261295-s002.pdf]

Data Sharing Statement

Shadyab. Plasma Phosphorylated Tau 217 and Incident Mild Cognitive Impairment and Dementia in Older Women. *JAMA Netw Open*. Published March 10, 2026. doi:10.1001/jamanetworkopen.2026.1295

Data
Data available: Yes
Data types: Deidentified participant data, Data (not involving human participants), Data dictionary
How to access data: De-identified data from the study and supporting documents can be made available after publication to researchers with investigator support, after approval of a proposal by the WHI Publications and Presentations Committee and with a signed data access agreement (see https://www.whi.org/doc/PP-policy.pdf). Please contact Aladdin Shadyab (ahshadya@health.ucsd.edu) and Linda McEvoy (linda.k.mcevoy@kp.org).
When available: With publication

Supporting Documents
Document types: None

Additional Information
Who can access the data: De-identified data from the study and supporting documents can be made available after publication to researchers with investigator support, after approval of a proposal by the WHI Publications and Presentations Committee and with a signed data access agreement (see https://www.whi.org/doc/PP-policy.pdf). Please contact Aladdin Shadyab (ahshadya@health.ucsd.edu) and Linda McEvoy (linda.k.mcevoy@kp.org).
Types of analyses: New or replication analyses for the purpose of academic research.
Mechanisms of data availability: After approval of a proposal and with a signed data access agreement
